# Trifluridine/tipiracil plus bevacizumab as a first‐line treatment for elderly patients with metastatic colorectal cancer (KSCC1602): A multicenter phase II trial

**DOI:** 10.1002/cam4.3618

**Published:** 2020-11-29

**Authors:** Eiji Oki, Akitaka Makiyama, Yuji Miyamoto, Masahiko Kotaka, Hirofumi Kawanaka, Keisuke Miwa, Akira Kabashima, Tomohiro Noguchi, Kotaro Yuge, Tomomi Kashiwada, Koji Ando, Mototsugu Shimokawa, Hiroshi Saeki, Yoshito Akagi, Hideo Baba, Yoshihiko Maehara, Masaki Mori

**Affiliations:** ^1^ Department of Surgery and Science Graduate School of Medical Sciences Kyushu University Fukuoka Japan; ^2^ Department of Hematology/Oncology Japan Community Healthcare Organization Kyushu Hospital Kitakyushu Japan; ^3^ Cancer Center Gifu University Hospital Gifu Japan; ^4^ Department of Gastroenterological Surgery Kumamoto University Graduate School of Medicine Kumamoto Japan; ^5^ Gastrointestinal Cancer Center Sano Hospital Kobe Japan; ^6^ Department of Surgery National Hospital Organization Beppu Medical Center Beppu Japan; ^7^ Department of Surgery Kurume University Hospital Kurume Japan; ^8^ Department of Surgery National Hospital Organization Oita Medical Center Oita Japan; ^9^ Department of Surgery Imakiire General Hospital Kagoshima Japan; ^10^ Department of Surgery Social Insurance Tagawa Hospital Tagawa Japan; ^11^ Department of Internal Medicine Division of Hematology, Respiratory Medicine and Oncology Faculty of Medicine Saga University Saga Japan; ^12^ Department of Biostatistics Yamaguchi University Yamaguchi Japan; ^13^ Department of Gastroenterological Surgery Gunma University Graduate School of Medicine Maebashi Japan; ^14^ Kyushu Central Hospital of the Mutual Aid Association of Public School Teachers Fukuoka Japan

**Keywords:** bevacizumab, colorectal cancer, elderly, thymidine phosphorylase inhibitor, Trifluridine

## Abstract

**Background:**

A previous Phase I/II study demonstrated that TAS‐102 (trifluridine/tipiracil [FTD/TPI]) plus bevacizumab (Bev) has encouraging efficacy and controllable safety for patients with previously treated metastatic colorectal cancer. Therefore, we designed for assessing the efficacy and safety of FTD/TPI plus Bev in elderly patients with previously untreated metastatic colorectal cancer.

**Methods:**

This is a multicenter, single‐arm Phase II study included patients ≥70 years old with previously untreated, unresectable metastatic colorectal cancer. Treatment consisted of FTD/TPI plus Bev given every 4 weeks. The primary endpoint was progression‐free survival (PFS), assuming a null hypothesis of a PFS of 5 months. The secondary endpoints were the overall survival (OS), overall response rate (ORR), and adverse events (AEs).

**Results:**

Between 5 January 2017 and 13 March 2018, 39 patients were enrolled from 18 institutions. The median patient age was 76.0 years (range, 70–88); the ECOG‐PS was 0 in 24 patients and 1 in 15 patients. The median PFS was 9.4 months as a primary endpoint, and the median OS was 22.4 months. The ORR was 40.5% and the disease control rate was 86.5%. Grade 3–4 AEs included neutropenia (71.8%), leukopenia (51.3%), anorexia (15.4%), febrile neutropenia (10.3%), and fatigue (10.3%).

**Conclusions:**

FTD/TPI plus Bev is an effective and well‐tolerated regimen for elderly patients with previously untreated metastatic colorectal cancer. Capecitabine/bevacizumab can be selected as a subsequent maintenance therapy without irinotecan and oxaliplatin because FTD/TPI has no cross‐resistance with 5‐fluorouracil.

Clinical trial registration: UMIN clinical trials registry (UMIN000025241).

## Graphical abstract

We aimed to assess the efficacy and safety of FTD/TPI plus Bev in elderly patients with previously untreated metastatic colorectal cancer. The median PFS was 9.4 months as a primary endpoint, the median OS was 22.4 month, ORR was 40.5% and AEs were manageable. The combination of FTD/TPI plus Bev is an effective and well‐tolerated regimen for elderly patients with previously untreated metastatic colorectal cancer.

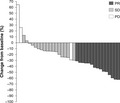

## 1 INTRODUCTION

Combination chemotherapy of FOLFOX (fluorouracil, folinate, and oxaliplatin), FOLFIRI (fluorouracil, folinate, and irinotecan), or FOLFOXIRI (fluorouracil, folinate, oxaliplatin, and irinotecan) is widely used in patients with colorectal cancer, combined with a biological agent, based on its high efficacy and acceptable toxicity.^1^ However, colorectal cancer is generally a disease of aged people, and the rapid aging of societies in many developed countries means that combinations of cytotoxic agents are often not tolerated in the majority of patients.^2^, ^3^ Therefore, bevacizumab (Bev) plus capecitabine was investigated in a Phase III trial as an alternative therapeutic option in elderly patients with metastatic colorectal cancer,^4^ particularly for those in whom first‐line oxaliplatin‐ or irinotecan‐based combination regimens are unsuitable. Thus, several guidelines recommend fluorouracil monotherapy or capecitabine with Bev as a first‐line treatment for patients with advanced colorectal cancer for whom more intensive treatment is inappropriate.^5^, ^6^, ^7^, ^8^

Trifluridine/tipiracil (TAS‐102 or FTD/TPI) is an orally administered combination drug of a nucleoside analog (FTD) and a thymidine phosphorylase inhibitor (TPI) at a molar ratio of 1:0.5. FTD is the active cytotoxic component of drug, and TPI prevents rapid degradation of FTD to its inactive form by thymidine phosphorylase.^9^, ^10^ FTD/TPI was established as a third‐line treatment for metastatic colorectal cancer following the findings of the international Phase III RECOURSE study, which reported a significant benefit for FTD/TPI in terms of overall survival (OS) compared to placebo, and an acceptable safety profile.^11^, ^12^

In preclinical studies, FTD/TPI in combination with Bev displayed better activity against colorectal cancer xenografts compared to FTD/TPI alone.^13^ In addition, a Phase I/II study demonstrated that FTD/TPI +Bev has favorable activity for patients with metastatic colorectal cancer who are refractory to several therapies.^14^ Indeed, FTD/TPI +Bev has been already used in many clinical settings in Japan, where it has been shown to be more effective than FTD/TPI monotherapy.^15^ Two randomized Phase II studies have been conducted worldwide, in which better survival was shown compared to FTD/TPI monotherapy or capecitabine +Bev.^16^, ^17^ As a result, two randomized Phase III studies for first‐line and second‐line treatments are currently being conducted.^18^, ^19^

Elderly people with colorectal cancer require chemotherapy which has a low incidence of AEs and enables them to maintain a good quality of life. FTD/TPI is an oral combination therapy with toxicity that is mild and well tolerated. Since non‐hematological adverse events (AEs) are uncommon following FTD/TPI treatment, the addition of Bev to this regimen may be appropriate for use in elderly patients. Therefore, we assessed the efficacy and safety of FTD/TPI +Bev for elderly patients with previously untreated metastatic colorectal cancer as Phase II study.

## 2 PATIENTS AND METHODS

### 2.1 Study design

This investigator initiated multicenter, open‐label, single‐arm Phase II trial was conducted at 18 institutions in Japan. The primary endpoint was progression‐free survival (PFS), which was assessed by the Central Review Committee. A null hypothesis of PFS was assumed 5 months as the PFS of capecitabine monotherapy arm of AVEX trial was 5.1 (95% CI 4.2–6.3)month.^4^ The secondary endpoints for efficacy included the overall response rate (ORR), OS, and safety. The Central Review Committee consisted of three independent oncologists who judged efficacy by imaging according to the Response Evaluation Criteria in Solid Tumors (RECIST) guideline, and without any other clinical information. Patients were examined at 6‐week intervals to evaluate the target lesions. Toxicity was assessed according to the NCI‐CTC version 4.0.

### 2.2 Patients

Patients aged 70 years and older were included, and eligible patients were divided into two groups, as either fit or vulnerable. The fit group comprised patients who were able to receive the standard therapy administered to young patients, but who decided not to receive oxaliplatin‐ or irinotecan‐containing regimens. The vulnerable group comprised patients who were unable to receive the standard therapy administered to young patients, but could receive alternative treatments. Other inclusion criteria were (a) metastatic adenocarcinoma of the colon or rectum (not anal canal cancer); (b) no history of previous chemotherapy, radiation therapy, or immunotherapy excluding adjuvant chemotherapy; (c) the presence of measurable disease (RECIST version 1.1); (d) Eastern Cooperative Oncology Group (ECOG) performance status (PS) of 0 or 1; and (e) adequate organ function. *RAS* genetic screening was not mandatory, but the status and method were collected if the *RAS* test was performed.

### 2.3 Procedures

Bev was administered intravenously at a dose of 5 mg/kg on days 1 and 15. FTD/TPI was administered orally at a twice‐daily dose of 35 mg/m^2^ on days 1–5 and 8–12. Treatment courses were repeated every 28 days until disease progression, patient refusal, or the unacceptable toxicity. FTD/TPI dose reductions were specified in the protocol if the neutrophil count was <1000/mm^3^, platelet count was <50,000/mm^3^, or grade 3 or worse non‐hematological AEs occurred. Under these circumstances, the FTD/TPI dose was reduced to 30 (−1 level), 25 (−2 level), or 20 (−3 level) mg/m^2^ after the first, second, and third occurrence of such events, respectively. All treatments were delivered by participate site investigator physicians.

### 2.4 Statistical analysis and Outcomes

Sample size is based on the nonparametric estimate of the survival distribution with PFS as primary endpoint. Assuming a null hypothesis of PFS of 5 months and an alternative hypothesis of PFS of 9 months, with a one‐sided type I error of 0.1 and type II error of 0.2, it was necessary to enroll a minimum of 32 patients. Considering dropouts and withdrawals, enrollment of over 35 patients was planned. Median survival was estimated using the Kaplan–Meier method, and the 95% CIs were calculated based on the Brookmeyer and Crowley method. Response was calculated based on RECIST 1.1. The ad hoc subgroup analysis was performed for geriatric assessment, ras, and response. All data were stored by the data center of Clinical Research Support Center Kyushu. Patients who completed at least one treatment course were included in all safety and efficacy analyses. All statistical analyses were performed using SAS version 9.4 (SAS Institute).

## 3 RESULTS

### 3.1 Patient backgrounds and treatment

Between 5 January 2017 and 13 March 2018, 39 patients were enrolled from 18 institutions in Japan. Two patients were excluded due to absence of CT, and 37 patients were included finally in the efficacy analysis. All 39 enrolled patients were followed over 2 years except cases of death and included in the safety analysis (Figure S1), and the baseline patient characteristics are shown in Table 1. The median age of the 39 patients was 76.0 years (range, 70–88), and the ECOG PS was 0 in 24 patients (61.5%) and 1 in 15 patients (38.5%). Ras was found to be wild type in 10 patients (25.6%) and mutant in 23 patients (59.0%), whereas the Ras status was not determined in 6 patients (15.4%). The numbers of fit and vulnerable patients were 23 and 16, respectively.

**TABLE 1:** Patient characteristics

Variables		All enrolled patients	FAS^a^
		*n* = 39	*n* = 37
		(%)	(%)
Age	Median (Min–Max)	76.0 (70‐88)	76.0 (70‐88)
Sex	Male	17 (43.6)	15 (40.5)
	Female	22 (56.4)	22 (59.5)
ECOG^a^ performance status	0	24 (61.5)	23 (62.2)
	1	15 (38.5)	14 (37.8)
Geriatric assessment	Fit	23 (59.0)	22 (59.5)
	Vulnerable	16 (41.0)	15 (40.5)
Primary tumor site	Right	18 (36.1)	17 (45.9)
	Left (Inc. rectum)	21 (53.9)	20 (54.1)
Pathology	Tub	32 (82.1)	30 (81.1)
	Por	5 (12.8)	5 (13.5)
	Muc	2 (5.1)	2 (5.4)
Ras	Wild type	10 (25.6)	10 (27.0)
	Mutant	23 (59.0)	21 (56.8)
	Not investigated	6 (15.4)	6 (16.2)

^a^ Abbreviations: FAS, full analysis set; ECOG, Eastern Cooperative Oncology Group.

The 39 patients received a total of 254 treatment courses (median, 6.5 courses; range, 3–9 courses), and the median time to treatment discontinuation (TTD) was 166 (Min–Max: 14–507) days. Fit/vulnerable patients with median TTD were 168 (min–max: 14–507)/160 (min–max: 68–399) days, Ras wild/mutant patients with median TTD were 168 (min–max: 56–507)/136 (min–max: 27–399) days. The major reasons for treatment discontinuation in 38 patients (one patient is still undergoing treatment) were disease progression (*n* = 21), AEs (*n* = 9) (Table S1), surgery (*n* = 4), and patient refusal (*n* = 4). The mean relative dose intensities were 77.5% (95% CI = 70.7–84.2) for FTD/TPI and 79.4% (95% CI = 73.7–85.0) for Bev. The cutoff date for the primary endpoint analysis was 22 January 2020.

### 3.2 Efficacy analysis

**FIGURE 1:**
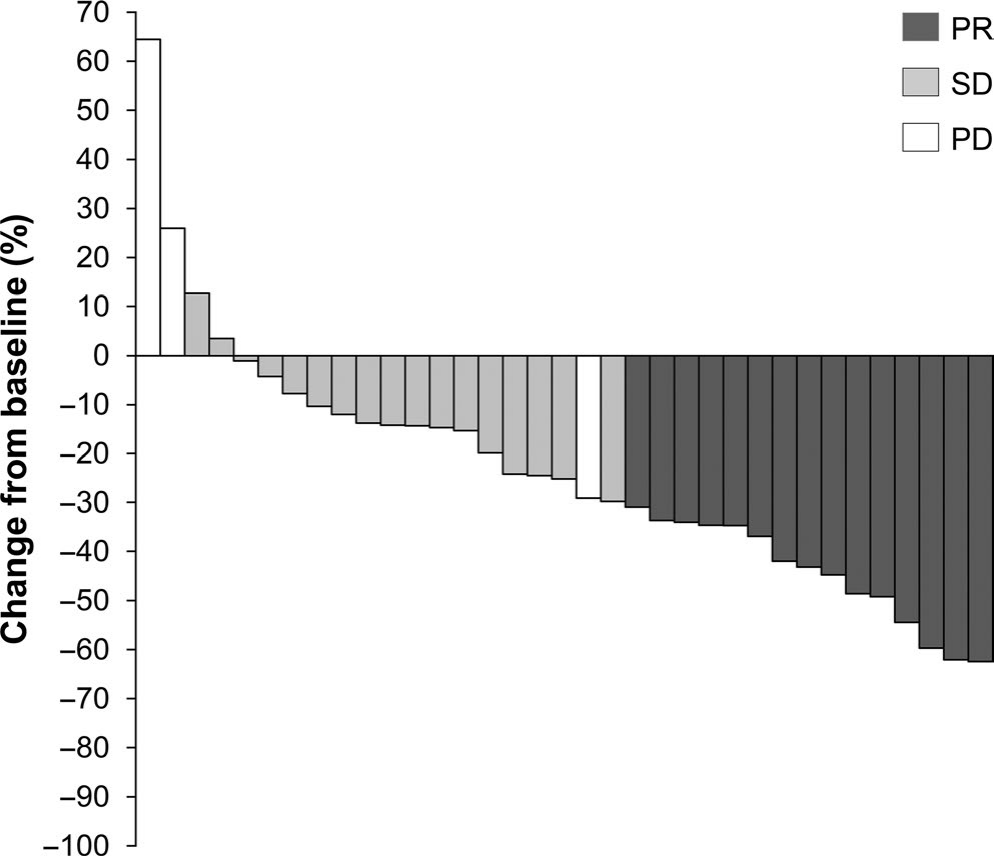
Waterfall plot

The ORR was decided by the Central Review Committee in 37 patients. The partial response (PR) in 15 patients; hence, the ORR (complete response [CR] +PR) was 40.5% (95% CI = 24.7–57.9) (Table 2). Figure 1 shows a waterfall plot of response rate. Furthermore, 17 patients (45.9%) had stable disease (SD), and the disease control rate (CR + PR + SD) was 86.5% (95% CI = 71.2–95.5). The response rate of the fit patients was 9/22 (40.9%), and that of vulnerable patients was 6/15 (40.0%), (*p* = 1.0000).

**TABLE 2:** Response rate

	*n*	%
CR^a^	0	0
PR^a^	15	40.5
SD^a^	17	45.9
PD^a^	3	8.1
NE^a^	2	5.4
ORR^a^	15	40.5 (95% confidence interval = 24.7‐57.9)
DCR^a^	32	86.5

^a^ Abbreviations: CR, complete response; PR, partial response; SD, stable disease; PD, progressive disease; NE, not evaluated; ORR, overall response rate; DCR, disease control rate

**FIGURE 2:**
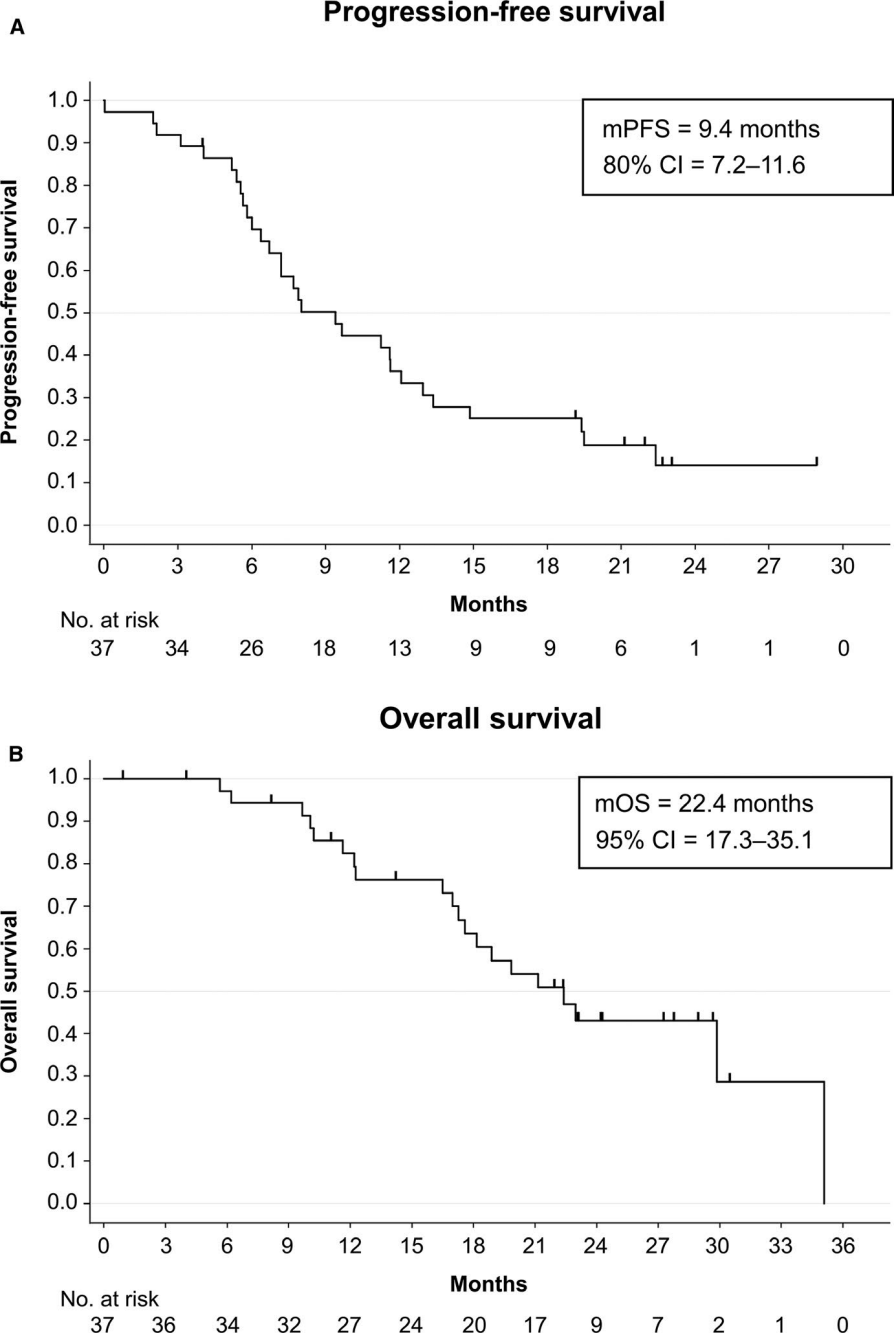
(A) Progression‐free survival. (B) Overall survival

The median follow‐up period was 18.9 months as of 22 January 2020. The median PFS was 9.4 months (80% CI = 7.2–11.6) (Figure 2A) (95% CI = 7.2–11.6) as the primary endpoint, and the median OS was 22.4 months (95% CI = 17.3–35.1) (Figure 2B).

In patients with wild‐type Ras, the median PFS was 6.2 months (95% CI = 2.0–19.4), versus 9.7 months (95% CI = 7.2–12.1) in patients with mutant Ras (HR, 0.841; 95% CI = 0.364–1.944; *p* = 0.6843). Among fit patients, the median PFS was 10.4 months (95% CI = 6.0–13.4) compared to 7.2 months (95% CI = 5.4–14.9) in vulnerable patients (HR, 0.694; 95% CI = 0.336–1.435; *p* = 0.3215) (Figure S2).

In total, 23 patients (62.2%) received second‐line chemotherapy (Table S2), and the main second‐line regimen among the 23 patients that required subsequent treatment was capecitabine+Bev or S‐1+Bev (seven patients; 30.5%). In addition, 10 patients were treated with oxaliplatin‐containing therapy.

### 3.3 Toxicity

Table 3 summarized the AE in this study. Grade 3–4 AEs included neutropenia (71.8%), leukopenia (51.3%), anorexia (15.4%), and febrile neutropenia (10.3%), while the major non‐hematological AEs were hypertension (23.1%) and fatigue (10.3%).

**TABLE 3:** Adverse events according to CTCAE version 4.0

Variables	^a^CTCAE version 4.0, *n* = 39 (%)					
	Grade 1	Grade 2	Grade 3	Grade 4	≥Grade 3	All grade
Neutropenia	2 (5.1)	6 (15.4)	14 (35.9)	14 (35.9)	28 (71.8)	36 (92.3)
Leukopenia	1 (2.6)	14 (35.9)	17 (43.6)	3 (7.7)	20 (51.3)	35 (89.7)
Anemia	7 (17.9)	24 (61.5)	6 (15.4)	2 (5.1)	8 (20.5)	39 (100.0)
Thrombocytopenia	23 (59.0)	6 (15.4)	1 (2.6)	0 (0.0)	1 (2.6)	30 (76.9)
Hypertension	2 (5.1)	17 (43.6)	9 (23.1)	0 (0.0)	9 (23.1)	28 (71.8)
Anorexia	16 (41.0)	10 (25.6)	6 (15.4)	0 (0.0)	6 (15.4)	32 (82.1)
Febrile neutropenia	0 (0.0)	0 (0.0)	4 (10.3)	0 (0.0)	4 (10.3)	4 (10.3)
Fatigue	11 (28.2)	6 (15.4)	4 (10.3)	0 (0.0)	4 (10.3)	21 (53.8)
ALT increased	6 (15.4)	1 (2.6)	2 (5.1)	0 (0.0)	2 (5.1)	9 (23.1)
Hyponatremia	17 (43.6)	0 (0.0)	1 (2.6)	1 (2.6)	2 (5.1)	19 (48.7)
Hyperkalemia	13 (33.3)	1 (2.6)	3 (7.7)	0 (0.0)	3 (7.7)	17 (43.6)
Diarrhea	11 (28.2)	2 (5.1)	2 (5.1)	0 (0.0)	2 (5.1)	15 (38.5)
TB^a^ increased	4 (10.3)	1 (2.6)	1 (2.6)	0 (0.0)	1 (2.6)	6 (15.4)
AST^a^ increased	18 (46.2)	0 (0.0)	0 (0.0)	1 (2.6)	1 (2.6)	19 (48.7)
Hypoalbuminemia	27 (69.2)	11 (28.2)	1 (2.6)	0 (0.0)	1 (2.6)	39 (100.0)
Hypokalemia	12 (30.8)	0 (0.0)	1 (2.6)	0 (0.0)	1 (2.6)	13 (33.3)
Fever	5 (12.8)	2 (5.1)	1 (2.6)	0 (0.0)	1 (2.6)	8 (20.5)
Vomiting	11 (28.2)	2 (5.1)	1 (2.6)	0 (0.0)	1 (2.6)	14 (35.9)

^a^ Abbreviations: CTCAE, common terminology criteria for adverse events; T.B, total bilirubin; AST, aspartate aminotransferase

## 4 DISCUSSION

Standard therapy for unresectable colorectal cancer has been established in recent decades, and the therapeutic strategy has been continually updated^1^ Similarly to many cancers, colorectal cancer is generally a disease of elderly people. However, the vast majority of clinical studies are not designed for elderly patients. In addition, we do not have sufficient information regarding the results of chemotherapy in elderly patients. Based on the idea that elderly people cannot tolerate standard intensive chemotherapy,^3^ many guidelines recommend specific regimens for elderly or vulnerable patients.^1^, ^5^, ^6^, ^7^, ^8^ As a result, capecitabine +Bev has been widely used, and its efficacy was confirmed in a Phase III clinical study of elderly patients with colorectal cancer.^4^ Furthermore, subset analysis of the RECOUSE and a recent large observational study conducted in the USA indicated the efficacy and safety of FTD/TPI monotherapy in patients ≥65 years was similar to that reported in patients <65 years.^20^, ^21^In addition, a Japanese observation study showed that FTD/TPI +Bev demonstrated good efficacy in patients aged ≥65 years.^15^ Therefore, we expected good efficacy and safety even though our study targeted patients over 70 years old.

In our study, FTD/TPI +Bev exhibited good efficacy and acceptable safety in elderly patients; this finding was similar to that of capecitabine +Bev, in which 280 patients were randomized to capecitabine alone and capecitabine +Bev arms, and the PFS of capecitabine +Bev arm was 9.1 months (95% CI = 7.3–11.4).^4^ FTD/TPI +Bev has been previously evaluated as a first‐line therapy for vulnerable patients, and the TASCO‐1 study reported a median PFS of 9.23 months (95% CI = 7.59–11.56), which was in line with our result of 9.4 months.^17^ Thus, the two studies report similar efficacy and acceptable safety despite having different eligibility criteria. In addition, our results demonstrated that FTD/TPI +Bev produced a better response rate in the first‐line setting than in the third‐line or later in other clinical trials^14^, ^16^ in which the response rate was below 4%. Indeed, the response rate of 40.5% in the current study was even better than that of the AVEX trial,^4^ which reported a response rate of 19% for capecitabine +Bev in the first‐line treatment of elderly patients.

In the FTD/TPI +Bev regimen, the major treatment‐related grade 3 or worse AEs, excluding hematological events, included hypertension and fatigue. These AEs were acceptable because many symptoms can be managed in elderly people by oral therapy without hospitalization. In the AVEX study, which described the efficacy of capecitabine +Bev, grade 3 or worse hand–foot skin reactions (HFS) were observed in 16% of elderly patients with colorectal cancer.^4^ Conversely, HFS was not observed in patients treated with FTD/TPI +Bev. Moreover, in patients treated with FTD/TPI +Bev in the third or later lines, Kuboki et al. reported grade 3 or worse hypertension and anorexia in 8% and 4% of patients, respectively^14^; this rate was lower than that reported in the current study. In our study, the rate of grade 3 or worse hypertension and anorexia was 23.1% and 15.4%, respectively, although the rate of grade 3 or worse neutropenia was similar to that observed in our trial. The incidence of non‐hematological AEs might be higher in elderly patients with this regimen; this point must be considered when interpreting these results.

The treatment sequence is an important element of our trial.^4^ We previously showed that 5‐FU‐based chemotherapy is effective even in tumors becoming refractory to FTD after trifluridine/tipiracil treatment.^22^ Since FTD/TPI does not have cross‐resistance with 5‐fluorouracil,^10^, ^13^, ^23^ capecitabine/Bev can be selected as a subsequent therapy. This sequence can permit long‐term maintenance therapy without irinotecan and oxaliplatin. In the AVEX study, 37.8% patients required a second‐line chemotherapy regimen, while in our trial, 23 patients (62.2%) received second‐line chemotherapy. In fact, six patients were treated with capecitabine/Bev and one patient was treated with S‐1/Bev after the protocol in our trial (Table S2). Thus, long‐term maintenance is achievable if both FTD/TPI +Bev and capecitabine +Bev regimens are used.

The limitation is the difficulty in comparing the ORR and PFS with those obtained in other therapies, since this study was single‐arm Phase II study. Therefore, the ORR may have been overestimated. Careful comparison with the results of similar studies^15^, ^20^, ^21^ may provide suggestive evidence in support of an improved ORR with this combined drug regimen for the first‐line treatment of elderly patients in future studies.

In conclusion, we achieved our initial goal to evaluate the safety and efficacy of FTD/TPI +Bev in elderly patients. The AEs were mild, and the ORR was higher than expected; thus, FTD/TPI +Bev can be considered as a suitable candidate regimen for the first‐line treatment of elderly patients in future Phase III studies.

### 4.1 Ethics approval

We conducted this trial in accordance with the Declaration of Helsinki and Ethical Guidelines. The protocol was approved by the Institutional Review Boards of all participating institutions. UMIN clinical trials registry (UMIN000025241).

## 5 CONFLICT OF INTEREST STATEMENT

Eiji Oki has received honoraria for lecturing from Taiho Pharmaceutical Co., Ltd.; Yakult Honsha Co., Ltd.; Merck Serono; Takeda Pharmaceutical Co., Ltd.; and Chugai Pharmaceutical Co., Ltd. Akitaka Makiyama reports personal fees from Lily; personal fees from Chugai; and personal fees from Takeda, outside the submitted work. Masahito Kotaka reports other from Chugai Pharmaceutical Co., Ltd.; other from Yakult Honsha Co., Ltd.; other from Takeda Pharmaceutical Company Limited; other from Merck Biopharma Co., Ltd.; and other from Taiho Pharmaceutical Co., Ltd., outside the submitted work. Hideo Baba reports grants, personal fees, and nonfinancial support from Taiho Pharmaceutical Co., Ltd.; grants and nonfinancial support from Merck Biopharma Co., Ltd., during the conduct of the study; grants, personal fees, and nonfinancial support from Ono Pharmaceutical Co., Ltd.; grants, personal fees, and nonfinancial support from Eli Lilly Japan K.K.; grants and personal fees from Takeda Pharmaceutical Co., Ltd.; grants and personal fees from Chugai Pharmaceutical Co., Ltd.; grants from Shionogi & Co., Ltd.; grants from Covidien Japan Inc.; grants and nonfinancial support from Yakult Honsha Co., Ltd.; grants from Shin Nippon Biomedical Laboratories, Ltd.; grants from Novartis‐pharma K.K.; grants from Toyama Chemical Co., Ltd.; and grants and nonfinancial support from Johnson & Johnson K.K., outside the submitted work. Masaki Mori reports grants, personal fees, and nonfinancial support from Taiho Pharmaceutical Co., Ltd. The other authors have no conflicts of interest to declare. This work has been previously presented and published in abstract form as follows: ‐ Oki E., et al. Trifluridine/tipiracil plus bevacizumab in elderly patients with previously untreated metastatic colorectal cancer (KSCC1602): A multicenter, Phase II clinical trial. J Clin Oncol. 2019; 37(15): https://doi.org/10.1200/JCO.2019.37.15_suppl.3548 ‐ Makiyama A., et al. Bevacizumab plus trifluridine/tipiracil in elderly patients with previously untreated metastatic colorectal cancer (KSCC 1602): A single‐arm, Phase II study. Ann Oncol. 2019; 30, Suppl. 5:234–235. https://doi.org/10.1093/annonc/mdz246.097

## Supporting information

Fig S1

Fig S2

Table S1‐S2

## Data Availability

The data of this study are available on request to the corresponding author. The data are not open to the public due to ethical restriction.
