# Erratum

**DOI:** 10.1002/cam4.3947

**Published:** 2021-05-14

In the article by Oki E et al, entitled “Trifluridine/tipiracil plus bevacizumab as a first‐line treatment for elderly patients with metastatic colorectal cancer (KSCC1602): A multicenter phase II trial,”^1^ Dr. Kotaka's first name has been corrected and his full name should read “Masahito Kotaka.”
