# Supplementary figures and images for: Trifluridine/tipiracil plus bevacizumab as a first‐line treatment for elderly patients with metastatic colorectal cancer (KSCC1602): A multicenter phase II trial
Source: Cancer Med. 2020 Nov 29;10(2):454–61. doi: 10.1002/cam4.3618 (PMC7877360; doi:10.1002/cam4.3618)

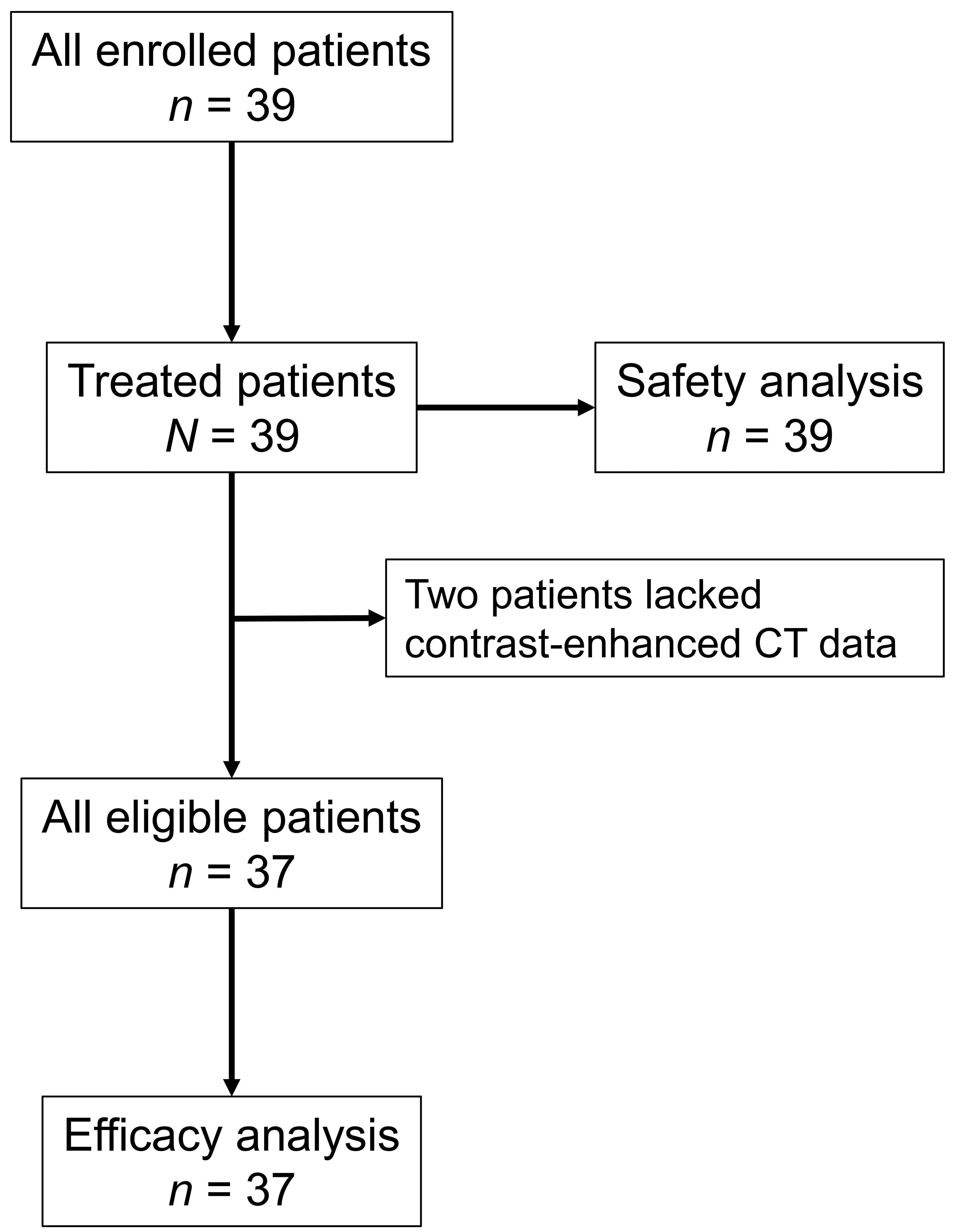

Supplement: Supplementary file 1 — Fig S1 [file CAM4-10-454-s001.tif]

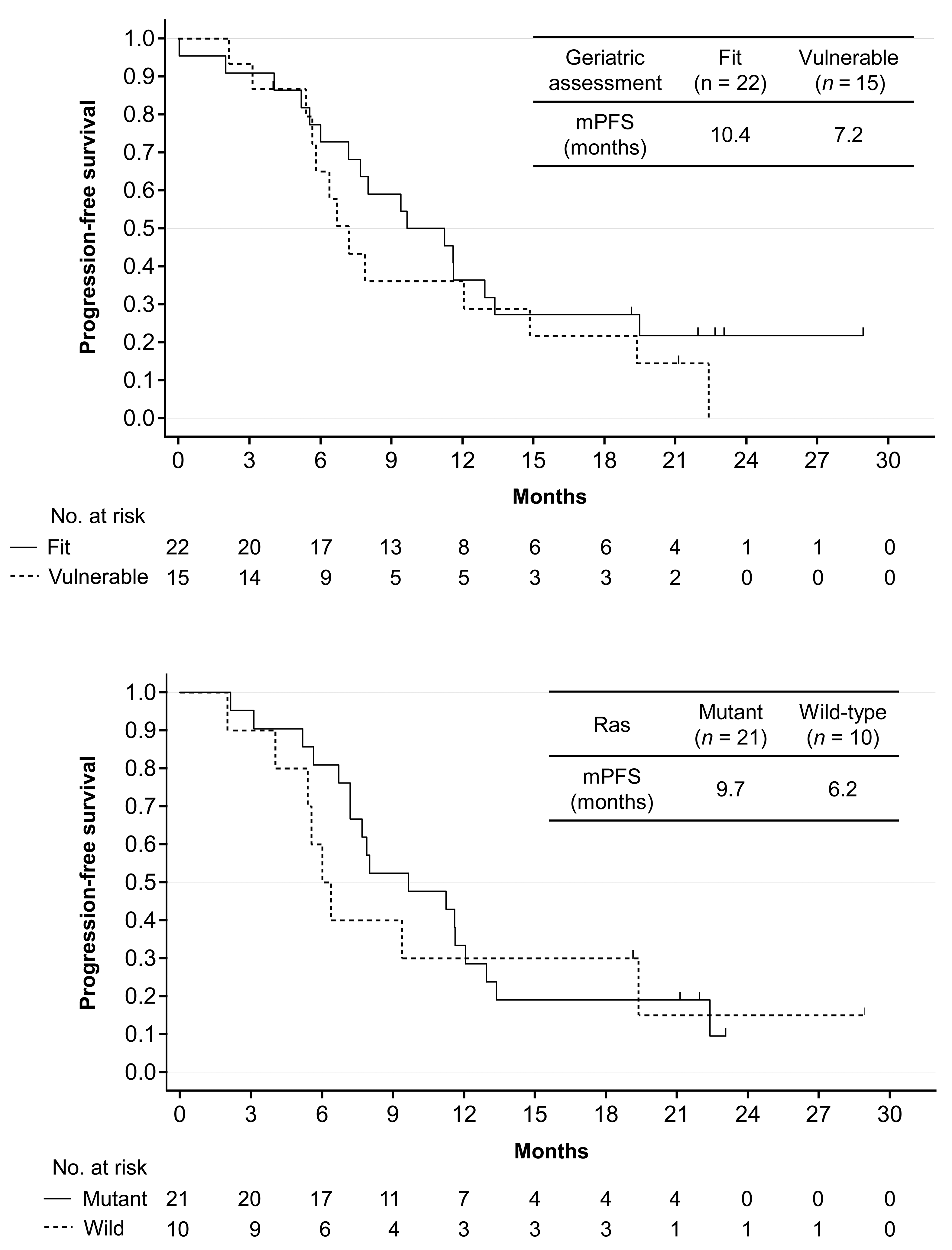

Supplement: Supplementary file 2 — Fig S2 [file CAM4-10-454-s002.tif]
