# Supplementary material for: Trifluridine/tipiracil plus bevacizumab as a first‐line treatment for elderly patients with metastatic colorectal cancer (KSCC1602): A multicenter phase II trial
Source: Cancer Med. 2020 Nov 29;10(2):454–61. doi: 10.1002/cam4.3618 (PMC7877360; doi:10.1002/cam4.3618)
Supplement: Supplementary file 3 — Table S1‐S2 [file CAM4-10-454-s003.docx]

**Supporting Information**

**Supporting Table 1.** Details of adverse events necessitating treatment discontinuation in each case

| Cases | Details of adverse events |
| --- | --- |
| 1 | Grade 4 neutropenia, Grade 4 leukopenia, Grade 3 anemia, Grade 3 febrile neutropenia |
| 2 | Grade 2 gastric ulcer, Grade 3 anemia |
| 3 | Grade 4 perforation of colon, Grade 3 leukopenia, Grade 3 anemia |
| 4 | Grade 4 anemia, Grade 3 neutropenia |
| 5 | Grade 4 duodenal ulcer |
| 6 | Grade 3 anorexia |
| 7 | Grade 1 vertigo |
| 8 | Grade 3 fatigue, Grade 3 anorexia |
| 9 | Grade 4 neutropenia |

**Supporting Table 2.** Subsequent therapy

| Subsequent therapy | | *N* |
| --- | --- | --- |
| Chemotherapy | |  |
|  | Capecitabine + BV, S-1 + BV | 7 |
|  | CAPOX + BV, SOX + BV | 7 |
|  | mFOLFOX, mFOLFOX6 + BV, mFOLFOX6 + Pmab | 3 |
|  | S-1 or Cape monotherapy | 2 |
|  | Other treatment | 4 |
|  | FOLFIRI + RAM |  |
|  | Pmab monotherapy |  |
|  | FTD/TPI |  |
|  | BV monotherapy |  |
|  | Total | 23 |
| Other treatment | | |
|  | Radiation | 2 |
|  | Surgery | 4 |
|  | No therapy | 8 |
| Total | | 37 |
| CAPOX, capecitabine/oxaliplatin; BV, bevacizumab; mFOLFOX6, 5-FU/leucovorin/oxaliplatin; Cape, capecitabine; Pmab, panitumumab | | |
